# A Multilevel Tailored Web App–Based Intervention for Linking Young Men Who Have Sex With Men to Quality Care (Get Connected): Protocol for a Randomized Controlled Trial

**DOI:** 10.2196/10444

**Published:** 2018-08-02

**Authors:** José A Bauermeister, Jesse M Golinkoff, Keith J Horvath, Lisa B Hightow-Weidman, Patrick S Sullivan, Rob Stephenson

**Affiliations:** ^1^ School of Nursing University of Pennsylvania Philadelphia, PA United States; ^2^ School of Public Health University of Minnesota Minneapolis, MN United States; ^3^ Institute of Global Health and Infectious Diseases University of North Carolina at Chapel Hill Chapel Hill, NC United States; ^4^ Rollins School of Public Health Emory University Atlanta, GA United States; ^5^ School of Nursing and the Center for Sexuality and Health Disparities University of Michigan Ann Arbor, MI United States

**Keywords:** HIV infections, AIDS, adolescent, internet, men who have sex with men, prevention, pre-exposure prophylaxis, awareness

## Abstract

**Background:**

HIV epidemic among young men who have sex with men (YMSM) is characterized by strong racial disparities and concerns about the availability and access to culturally appropriate HIV prevention and care service delivery. Get Connected, a Web-based intervention that employs individual- and system-level tailoring technology to reduce barriers to HIV prevention care (eg, HIV or sexually transmitted infection [STI] testing, pre-exposure prophylaxis [PrEP]), was developed for YMSM (age 15-24 years). This protocol details the design and procedures of a 2-phase project that includes mystery shopping and a randomized controlled trial (RCT) to test the efficacy of Get Connected among YMSM in Philadelphia, Atlanta, and Houston.

**Objective:**

The objective of mystery shopping is to examine the quality of HIV test counseling and PrEP-related referrals for YMSM within local HIV or STI testing sites. The objective of the RCT is to test the efficacy of Get Connected for increasing HIV-negative or HIV-unknown YMSM’s successful uptake of HIV prevention services (eg, routine HIV or STI testing), PrEP awareness, and likelihood to start PrEP (PrEP willingness), compared with those in the control condition, over a 12-month period.

**Methods:**

For Phase 1, we will create a master list of HIV and STI testing sites in each city. We will enroll and train 10-15 mystery shoppers per city; each testing site will be separately visited and assessed by 2 mystery shoppers. After each site visit, the mystery shoppers will complete a site evaluation to record their perceptions of various measures including lesbian, gay, bisexual, transgender, queer visibility and inclusivity, privacy and confidentiality, provider-patient interactions, and clinic environment. For Phase 2, we will enroll 480 YMSM for 12 months across the 3 iTech cities into a 2-arm prospective RCT. Participants randomized to the control condition are directed to the AIDSVu.org testing site locator. Participants randomized to the intervention condition will be granted access to a Web app with content tailored to their specific demographic characteristics (eg, age, race or ethnicity, location, and relationship status), HIV and STI risk behaviors (eg, HIV and STI testing history, substance use, communication with partners regarding status) and sociocultural context (eg, homelessness, incarceration). Study assessments will occur at enrollment and at 1, 3, 6, 9, and 12 months postenrollment.

**Results:**

Get Connected research activities began in September 2016 and are ongoing. To date, institutional review board (IRB) submission is complete and IRB authorization agreements are pending at several other universities.

**Conclusions:**

The deployment of Get Connected through a mobile-optimized Web app seeks to optimize the intervention’s acceptability, accessibility, availability, and long-term affordability among YMSM.

**Trial Registration:**

ClinicalTrials.gov (NCT03132415); https://clinicaltrials.gov/ct2/show/NCT03132415 (Archived by WebCite at http://www.webcitation.org/70j4gSFbZ)

**Registered Report Identifier:**

RR1-10.2196/10444

## Introduction

### Background

Young men who have sex with men (YMSM) now account for 72% of new HIV infections among people aged 13-24 years and 30% of all new infections among men who have sex with men (MSM) [1]. From 2008 to 2011, YMSM aged 13-24 years had the greatest percentage increase (26%) in diagnosed HIV infections [2], with approximately 93% of all diagnosed HIV infections from male-to-male sexual contact [2]. Among the drivers of the HIV epidemic among YMSM are large numbers of HIV-positive youth who are not virally suppressed or are not aware of their serostatus [3]. Increasing HIV testing among YMSM is thus a public health priority [4]. The success of the National HIV/AIDS Strategy’s test and treat approach rests on the ability to increase the number of YMSM receiving routine testing [5]. Getting tested is the cornerstone of almost all prevention approaches and the gateway to both biomedical prevention tools (eg, pre-exposure prophylaxis [PrEP]) and to HIV care for those who test positive.

Successful engagement in HIV prevention for HIV-negative youth (routine HIV testing, consistent condom use, PrEP adoption) requires that YMSM overcome a series of multilevel barriers at the individual (eg, risk awareness, self-efficacy to get tested), system (eg, costs, medical mistrust, lack of culturally competent care), and structural (eg, homelessness, stigma) levels [6-12]. Strategies to promote HIV or sexually transmitted infection (STI) status awareness among YMSM requires the creation of interventions that are culturally sensitive to the psychosocial needs of YMSM [13] and facilitate access to comprehensive sexual health services [14].

HIV prevention tools that are culturally and developmentally appropriate for YMSM are needed [1,7,15,16]. Web-based interventions are a promising mode of HIV or STI prevention given their ability to deliver responsive and interactive content specific to each user’s characteristics (ie, tailored content), with extended reach across geographic regions and increased convenience to access content at any time through tablets, laptops, and mobile phones. Furthermore, Web-based content can be updated to be contextually responsive over time, particularly as YMSM become sexually active, meet new partners, and engage in different risk behaviors. Collocating Web-based interventions is also important as YMSM often rank the Web as their top resource to access comprehensive sexual education, learn about their sexuality and sexual behavior, and meet partners [17].

Researchers and practitioners have sought to encourage routine HIV or STI testing by creating Web-based tools that provide the physical location of testing centers in a given geographic area (ie, testing locators). These testing locator interventions have demonstrated a wide reach when evaluated (eg, AIDS.gov test locator had over 16,000 searches and was adopted by over 100 websites in its first year [18]); however, there are limited data examining the quality and adequacy of these listed sites for YMSM. This is concerning for several reasons, as it is expected that testing agencies are youth and lesbian, gay, bisexual, transgender, questioning or queer (LGBTQ) friendly; however, there is little empirical evidence to support this assumption, and in fact evidence to support the contrary [19-23]. Using a mystery shopper methodology to evaluate the LGBTQ cultural competency and the quality of services offered in HIV and STI testing sites in Southeast Michigan (n=47 testing sites), we assessed the sites across 13 domains, including the clinic’s structural characteristics, and the test counselors’ compliance with the Centers for Disease Control and Prevention (CDC) HIV testing and counseling protocols [6]. After the mystery shopping assessment, we sent each site a letter describing our process and encouraged them to schedule a meeting with us to discuss the shoppers’ experiences at their agency. The agency staff was eager for the feedback and technical assistance, and 66% (31/47) of the sites requested to meet. In these meetings, we offered a packet of personalized results, summarizing how they compared on various quantitative indicators to other sites, and provided feedback from the open-ended portion of the evaluation. Several agencies noted that the report from the site evaluation would help focus their efforts and address identified areas for improvement.

### Theoretical Framework for Intervention

Building on the efficacy of the CDC’s project Connect Health Systems Intervention to link heterosexual adolescents to competent comprehensive sexual health care services [24], we developed Get Connected (GC), a Web-based brief intervention that employs individual- and system-level tailoring technology to reduce barriers to HIV prevention care (eg, HIV or STI testing, PrEP) for YMSM. The deployment of GC through a mobile-friendly Web app seeks to optimize the Web-based intervention’s acceptability, accessibility, availability, and long-term affordability among youth [4,7,25]. Using a consensus approach [26] to conceptualize health behavior change, the model guiding GC synthesizes the Integrated Behavioral Model [27] and Self-Determination Theory [28,29] as the theoretical underpinnings of our intervention. Consistent with these theories [30,31], GC content follows motivational interviewing principles [31-33] by focusing on resolving ambivalence about HIV prevention behaviors, increasing self-efficacy for change, and enhancing motivation moving toward action. GC participants are then recommended high-performing sites based on mystery shopper scores.

Participants in the pilot trial were randomized to receive a full GC intervention or an attention-control condition. Data [34] from this pilot randomized controlled trial (RCT; n=130 YMSM; age 15-24 years) indicated high acceptability and feasibility (80% retention, 104/130) for GC, and clinically meaningful effect sizes (ES) in self-efficacy to discuss HIV testing with partners (ES=0.50-0.64), trust in their providers (ES=0.33-0.35), reductions in number of sexual partners (ES=0.21), and HIV or STI testing behavior (ES=0.34; 30 participants tested for HIV or STIs) at the 30-day postintervention follow-up. Participants who received the GC intervention reported that the testing site information was more accurate than those in control condition. For all other acceptability items, the GC intervention was equally or slightly better received than the control condition. More than 90% (102/104, 92.3%) of participants reported that the GC intervention had been useful to identify a HIV or STI clinic that met their needs. We identified 1 incident HIV-positive case and 2 STI (herpes and chlamydia) cases over the 30-day study period.

As a step toward filling the current gap in efficacious Web-based interventions for HIV prevention and care among YMSM, we propose to implement and test the efficacy of GC 2.0 across 3 iTech cities heavily impacted by HIV: Philadelphia, Atlanta, and Houston. This protocol describes the methods for the testing of the intervention.

## Methods

### Trial Registration, Ethics, Consent, and Institutional Review Board Approval

The research and ethics presented in this study were approved by the IRB of the University of North Carolina at Chapel Hill (16-3183). A Certificate of Confidentiality has been obtained from the National Institute of Child Health and Human Development, and a waiver of parental consent or assent has been obtained for participants who are 15-17 years old. This study is also registered on ClinicalTrials.gov (NCT03132415).

### Phase 1: Mystery Shopping

#### Design

We will enroll mystery shopping participants (10-15 mystery shoppers per city) to conduct the mystery shopper assessment in 3 iTech cities: Philadelphia, Atlanta, and Houston. This approach follows best practices suggesting that youth involvement is vital when designing relevant and appropriate HIV interventions for the target population. We will work with iTech subject recruitment venues (SRV) in each city to recruit and enroll HIV-negative YMSM (age 18-24 years) who are interested in serving as mystery shoppers. We will apply a multimodal recruitment strategy, including ads in Web-based LGBTQ listservs, flyers in HIV or AIDS community-based organizations, local coffee shops and bars, college listservs, and Web-based advertisements on social media sites such as Facebook.

#### Mystery Shopper Participants

Eligible mystery shoppers are participants assigned male sex at birth and who currently identify as male; must be 18-24 years old (inclusive) at the time of screening; self-report as HIV-negative, speak and read English, live in Philadelphia, Atlanta, or Houston; must be able to travel to and from HIV or STI testing sites; report same-sex attraction; and have access to the internet via a computer or mobile phone.

#### Sample Size

We will recruit and enroll 10-15 mystery shoppers per city. Each participant can visit up to 10 unique testing sites in their city, with 2 mystery shoppers visiting each testing site, separately. Testing sites will be identified in collaboration with each city’s health department and by crosschecking sites with AIDSVu.org. We will employ a stratified purposive sampling strategy to ensure age and racial or ethnic diversity across mystery shoppers. Of the 10-15 mystery shoppers per city, 5-8 will be aged 18-20 years (2-3 Black or African American, 2-3 White, and 1-2 Hispanic or Latino) and 5-8 will be aged 21-24 years (2-3 Black or African American, 2-3 White, and 1-2 Hispanic or Latino).

#### Incentives

The mystery shoppers will receive a maximum of US $600: US $100 for attending the 1-day training session and US $50 for each testing site visit (10 maximum site visits).

#### Procedures

Once the mystery shoppers are consented and enrolled, they will attend a 1-day training at the iTech SRV where they will learn about the fundamentals of HIV or STI transmission, the guidelines and protocols surrounding HIV or STI testing and PrEP eligibility, and how to use the Web-based site assessment survey to evaluate their site visits. State-specific guidelines and policies will also be discussed in each city. Additionally, they will receive training to strengthen their self-efficacy to feel empowered as clients. Specifically, we will conduct role-plays with scenarios and interactions that might occur during a visit. We will underscore the importance of being well-versed in their rights and procedures and provide skills on how to respond to worst-case scenarios (eg, how to turn down any unwanted procedures), were they ever to occur. The mystery shoppers will be instructed to be honest about their sexual behaviors during their visits. By avoiding creating “personas” or “scripts,” shoppers will increase the social validity of the assessment and avoid arousing suspicion due to exaggerated or unrealistic scenarios.

The study staff will create and use a secure database to manage the mystery shoppers, site assignments, and testing schedules. Upon completion of the 1-day training, study staff will assign the mystery shoppers a specific day and time for their initial testing site visit. The mystery shoppers will report to the iTech SRV before each scheduled site visit to check in with a staff member and receive their site assignment. They will be loaned a mobile phone equipped with a car share app to use for travel to and from testing sites. All car share trips will be tracked and paid for by the study, so no transportation costs will be incurred by the mystery shoppers.

Once at the testing site, the mystery shoppers will state that they have no income, health insurance, or any proof of identification. In doing so, we will be able to ascertain whether these would be potential barriers to testing at a given location and ascertain the lowest possible fees that would be charged to YMSM. As in the original study [34], we will reimburse the mystery shoppers for any charges linked to their testing experiences. Upon completion of a testing visit, the mystery shoppers will use the mobile phone’s car share app to travel back to the iTech SRV. They will complete the site assessment survey on the mobile phone or on a computer at the iTech SRV. Mobile phones will be returned to the study staff upon return to the SRV.

The site assessment survey will record shoppers’ perceptions of their testing experience, specifically LGBTQ visibility, medical form inclusivity, clinic environment, privacy and confidentiality, PrEP information and dialogue, patient-provider relationship context, patient-provider counseling, safer sex education, perceived provider competency, and participant-provider interactions (Textbox 1). The mystery shoppers will also have the opportunity to leave qualitative feedback in an open text field if they wish to explain any of their responses or record any other information pertinent to their experience that the quantitative assessment did not already capture.

In addition to the site assessment survey, the mystery shoppers will complete a secure video chat session with study staff to discuss their testing experience and have the opportunity to share any adverse interactions. These video chat sessions will not be recorded; their purpose will be to ensure mystery shoppers’ safety and prevent subsequent mystery shoppers from being exposed to a site reported to be risky or unsafe (physically or emotionally). Following the video chat session, the mystery shoppers will be given their incentive for the visit and their next visit with study staff will be scheduled before leaving the iTech SRV.

#### Outcomes

The mystery shoppers’ site assessment scores will be aggregated for use in Phase 2 of the research activities: RCT to test the efficacy of GC. Specifically, the scores for each site will be averaged and embedded in the intervention condition of the GC Web app: when participants search for testing sites they will only see sites that rank in the top 50% for that city, sorted from highest to lowest ranking.

### Phase 2: Randomized Controlled Trial

#### Design

The research activities involve a 2-arm 12-month prospective RCT enrolling 480 HIV-negative or status-unaware YMSM (age 15-24 years). After assent or consent and completion of a baseline survey, YMSM will be randomized on a 1:1 basis to either the control or intervention condition (intervention, n=240; control, n=240). Participants randomized to the control condition will be directed to the AIDSVU.org testing site locator. While the provision of a test locator is a low intensity intervention, we felt that withholding referrals to testing and care services would be unethical given YMSM’s vulnerability to HIV and STIs. Furthermore, given the availability of search engines to locate HIV or STI testing sites, the test locator condition may be considered usual care. Nevertheless, by providing the existing testing site locator only, we will still be able to test the effect of GC (ie, user-tailored content focused on HIV or STI testing and PrEP referral and the linkage to high-quality agencies). Web-based study assessments are conducted every 3 months across the intervention and control conditions, with a total follow-up period of 12 months. At the end of RCT, we will make the intervention accessible to YMSM in the control condition.

#### Intervention

The GC intervention was developed by customizing content based on YMSM’s psychosocial and sexual profiles (eg, sociodemographics, HIV and STI testing history and testing motivations, recent sexual behavior, sources of support, self-reported values), as reported by participants’ answers to their baseline assessment. At the individual level, GC delivers tailored Web-based content specific to each user’s demographic characteristics (eg, age, race or ethnicity, location, relationship status), HIV and STI risk behaviors (eg, HIV and STI testing history, substance use, communication with partners regarding status), and sociocultural context (eg, homelessness, incarceration). GC also employs tailoring at the system level using mystery shopper scores. Participants across both conditions who have been tested will be asked to rate their visit at their quarterly follow-up assessment using the same mystery shopper criteria. Sites will receive biannual summaries, including the aggregated user reviews and brief technical assistance reports, to help sites understand their performance based on quality assurance evaluations from YMSM clients and to optimize service delivery, if needed.

**Figure 1:**
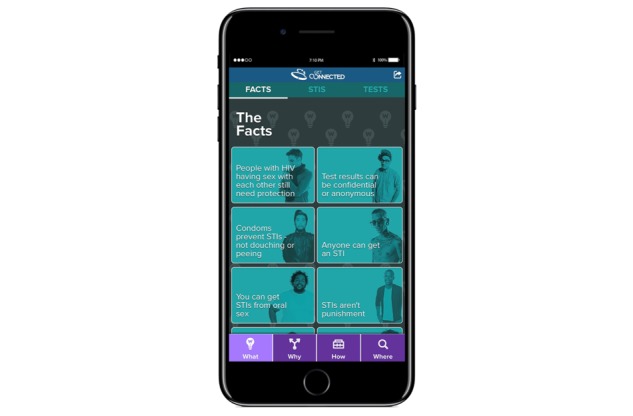
HIV and STIs (sexually transmitted infections) facts.

For the participants in the intervention condition, the tailored Web app has 4 sections of content: “What,” “Why,” “How,” and “Where.” The “What” section is split into 3 pages: “Facts,” “STIs,” and “Tests.” On each of those pages, topics are displayed in boxes that are randomly organized and open to display additional information if the user clicks or presses. The Facts page (Figure 1) displays boxes that contain general prevention facts (eg, “You won’t always know if someone has an STI.”) relevant to this population. On the STIs page, if a participant clicks “chlamydia,” they receive additional information about how it can be contracted, possible symptoms, testing options, and treatment options (if applicable). The Tests page displays boxes with each HIV or STI testing method (eg, blood test, swab test, urine test), and each box contains more specific information (eg, what STIs it tests for, steps for the test) upon click or press.

**Clinic and provider interaction visits to be recorded by the mystery shoppers.**

**Clinic characteristics**

Session speed (min)

LGBT visibility (Cronbach alpha=0.84)

- The clinic has symbols aimed at lesbian, gay, bisexual, transgender (LGBT) people (eg, equal sign, rainbow flag)
- The clinic has printed materials (eg, brochures) aimed at LGBT people
- The clinic has LGBT welcoming symbols

Medical forms (Cronbach alpha=0.59)

- The clinic uses LGBT-inclusive language on medical forms
- The clinic uses transgender-inclusive language on medical forms

Clinic environment (Cronbach alpha=0.76)

- The office staff were generally friendly
- The office staff were judgmental (Reverse coded)
- The office staff were not lesbian, gay, bisexual, transgender, questioning or queer (LGBTQ)–sensitive (Reverse coded)
- I felt uncomfortable in the waiting room (Reverse coded)
- The clinic used LGBT-affirming language when speaking to me

Privacy and confidentiality

- The clinic staff kept patient information confidential
- Interactions between clients and staff were kept private
- The provider explained confidentiality (either verbally or via a document)

Pre-exposure prophylaxis (PrEP)–specific indicators

- The clinic had information about PrEP
- The clinic offers PrEP or PrEP referrals

**Provider exchanges**

Relationship context (Cronbach alpha=0.89)

- The provider asked me about my sexual orientation
- The provider asked me about my relationship status
- The provider asked if I had experienced intimate partner violence

Counselling session (Cronbach alpha=0.76)

- The provider explored my motivation for testing
- The provider offered to help me set goals
- The provider offered to help me set action steps to meet safer sex goals
- The provider offered me risk reduction options
- The provider’s recommendations were valuable

Safer sex education (Cronbach alpha=0.88)

- The provider made sure I knew how to use a condom
- The provider helped me identify a condom that works for me
- The provider helped me identify a lube that works for me
- The provider discussed PrEP as a prevention strategy with me

Perceived provider competency (Cronbach alpha=0.65)

- The provider or test counsellor appeared knowledgeable about HIV and STIs
- The provider appeared knowledgeable about LGBTQ health issues

Negative provider interactions (Cronbach alpha=0.89)

- The provider made me feel comfortable (Reverse coded)
- I felt pressured by the provider to adopt specific risk reduction options
- The provider was judgmental about the kind of sex I have (eg, anal, receptive, or penetrative, etc)
- The provider was judgmental about how many partners I have had
- The provider was judgmental about how I met my partners

**Figure 2:**
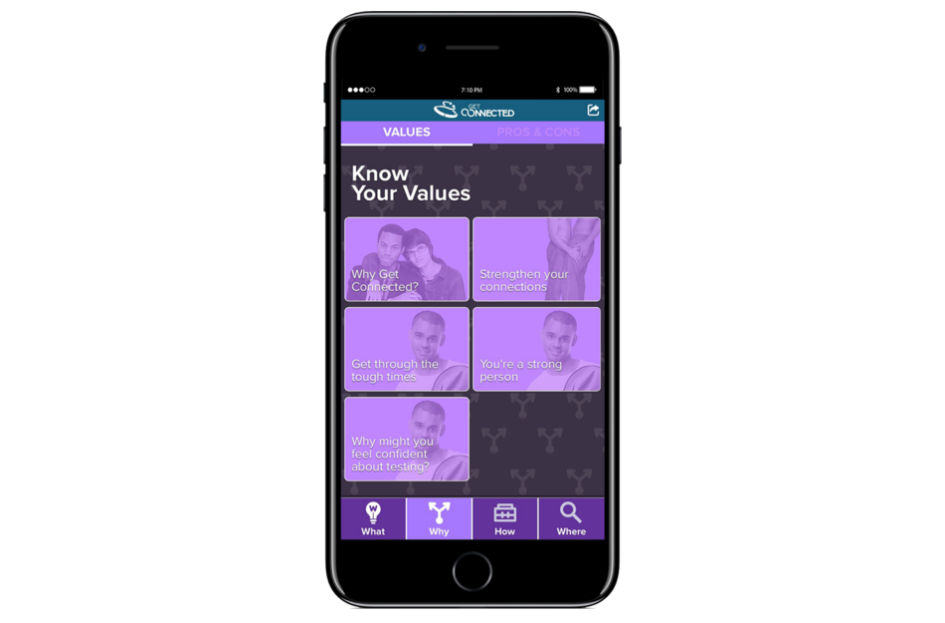
Values page.

The second section focuses on the “Why” on 2 pages: “Values” and “Pros and Cons.” The basic design and functionality is the same as described above in the “What” section. The Values page (Figure 2) encourages participants to assess their motivations, values, and strengths regarding HIV or STI testing. Reasons for getting tested are tailored to participants’ testing history (eg, “Never tested” versus “Tested for HIV, but not STIs”) in order to acknowledge their prior behaviors. Building on best practices, persuasive messages regarding the importance of linking to prevention services are then presented by linking participants’ values from the baseline survey (eg, being attractive, being religious, being sexy, being loved, being athletic, etc) to the desired outcomes. For example, a participant who indicated he valued being religious may see a message that says, “Finding strength in your faith. Your religious beliefs are important to you. Getting tested is one way to take care of, and honor, the body that you’ve been given. How might you draw on your faith to find strength to get tested?” The Pros and Cons page presents information on the perceived benefits and barriers of getting tested and of not getting tested.

**Figure 3:**
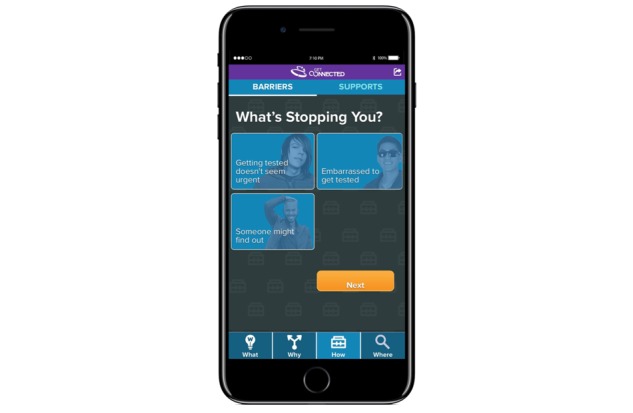
Barriers page.

The third section is about the “How” of testing and includes pages on potential “Barriers” to getting tested and “Supports” that may help a participant decide to get tested. Barriers (Figure 3) include issues like financial costs, social norms, and prioritization, which may affect participants’ desire to get tested for HIV or STIs. “Supports” has information on how their strengths and social support systems can help them make a choice about testing. Recognizing that barriers and supports may shift over time, content on these pages is tailored to identify the most recent barriers and supports as indicated by YMSM in their most recent survey.

**Figure 4:**
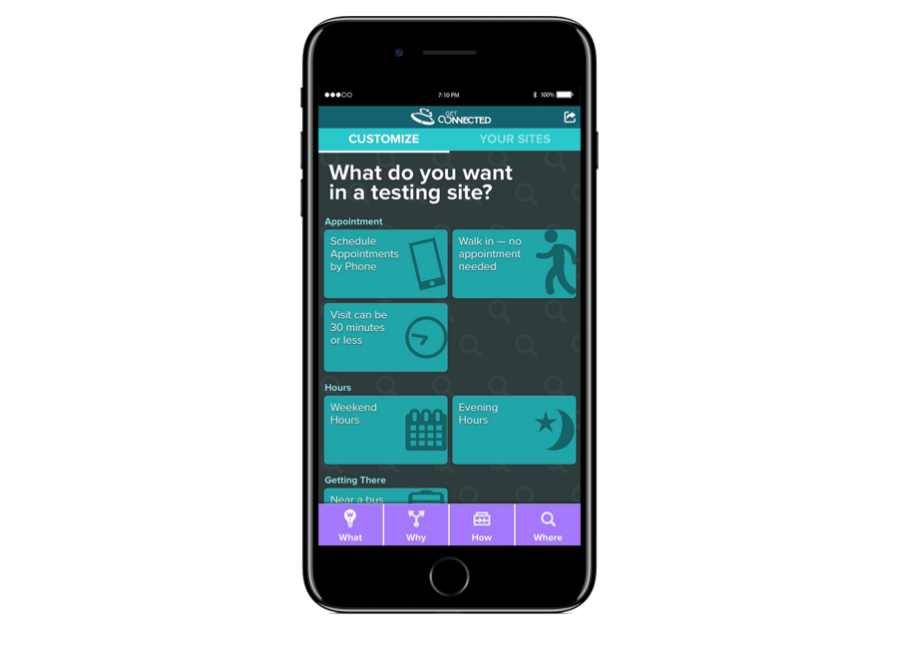
Customization page.

The final section is the “Where” of testing and includes a page where a user can “Customize” their search for nearby testing sites (Figure 4) and a “Your Sites” page that displays testing sites based on that customization. Participants can customize their search based on many clinic characteristics, including whether walk-in appointments are available, if they have weekend hours, and if they accept insurance. The “Your Sites” page is a listing of providers (including contact and location information) based on the participant’s customization selections. Testing sites are initially ranked using an algorithm that accounts for each site’s average mystery shopping scores. These scores are updated as participants get tested and rate sites over the 12-month study period. Participants can choose sites they may want to visit and then have the site information emailed or texted to them. Along with any site a participant emails or texts to themselves, they will be provided with 7 questions they can ask a provider during a testing visit. These questions were developed by the GC youth and community advisory boards and were found to be helpful to pilot trial participants when they encountered test counselors who were not perceived to be effective.

#### Participants

Eligible participants will be those assigned male sex at birth who currently identify as male, aged 15-24 years (inclusive) at the time of screening, have had consensual anal sex with another man in the past 6 months, self-report as HIV-negative or unsure of their HIV status, have access to a computer or mobile phone, can read and speak English, and live within the city limits of Philadelphia, Atlanta, or Houston.

#### Sample Size

Our target enrollment across both conditions is 480 participants (intervention, n=240; control, n=240). This number allows for 20% loss to follow-up rate and a final analytic sample of 400 YMSM across the 3 cities. Participants may continue the study even if they miss assessments intermittently over the data collection period. We will compare those who completed different follow-up assessments with those who did not based on key predictors from the baseline assessment to check for possible bias due to missing data and informative censoring. When appropriate, we will use expectation-maximization algorithm-based imputation methods in our analyses [35,36]. The primary outcomes for the proposed trial are successful uptake of HIV prevention services (eg, HIV or STI testing) and PrEP awareness and willingness. For HIV testing, we define power as correctly identifying the difference in the proportion of YMSM who engage in HIV testing 2 or more times at least 3 months apart during the 12-month follow-up period (“frequent tester”) in our treatment arm (GC) versus our control arm. For STI testing, we define it as receiving at least 1 STI test. For proportions (eg, HIV testing, PrEP awareness), our sample size calculations are based on a 2-sample test of proportions using a 2-sided significance level of 0.05.

In order to have 80% power to the intervention and control groups, we require at least 400 participants to find an absolute difference of 13% in cross-sectional analyses. Assuming a within-person correlation of 0.25, we can detect an 8.8% difference, indicating we have power to detect the smallest possible difference between arms. A less favorable within-person correlation of 0.75 allows us to detect an 11.3% difference. For mean differences across continuous outcomes (eg, PrEP willingness), our sample size calculations are based on a 2-sample *t* test, assuming equal variance using a 2-sided significance level of 0.05. We are able to detect a between-arm effect size difference of Cohen *d*=0.25 at the final follow-up time point at 80% power. For repeated measures analyses, assuming a within-person correlation of 0.25, we would be able to detect an effect size of 0.08. A less favorable within-person correlation of 0.75 allows us to detect an effect size of 0.11.

#### Incentives

Participants can earn up to US $155 total: Baseline survey=US $20, month 1 survey=US $20, month 3 survey=US $25, month 6 survey=US $30, month 9 survey=US $30, and month 12 survey=US $30.

#### Randomization

After assent or consent and completion of the baseline survey, YMSM will be randomized by city 1:1 to either the control or intervention condition (intervention, n=240; control, n=240) [37]. The stratified randomization process occurs upon completion of the baseline survey.

#### Outcomes

##### Primary Outcomes

The primary outcomes relate to the successful uptake of HIV prevention services among our sample of self-reported HIV-negative or serostatus-unaware YMSM. We have considered 3 prevention outcomes: HIV testing, STI testing, and PrEP awareness and willingness.

###### HIV Testing

The baseline survey will include questions on lifetime HIV testing history. Follow-up surveys will repeat the questions from the baseline and will also include questions on HIV testing in the prior 3-month period, including test results. The HIV testing outcome will be the proportion of YMSM tested for HIV 2 or more times at least 3 months apart in the 12-month follow-up period (“frequent tester”). As an additional analysis, we will also examine the proportions of participants who receive 1 HIV test.

###### Sexually Transmitted Infection Testing

The baseline survey will include separate questions on lifetime testing history of gonorrhea, chlamydia, and syphilis, respectively, as well as questions about ever having a genital exam, an anal pap smear, or a vaccination for Hepatitis A and B, human papilloma virus, and meningitis. Follow-up surveys will repeat the questions from the baseline but will ask about STI testing behavior in the prior 3-month period, including test results if a participant indicates they received a test. The STI testing outcome will be the proportion of YMSM tested for any STI 2 or more times, at least 3 months apart, in the 12-month follow-up period (“frequent tester”). As an additional analysis, we will also examine the proportions of participants who receive 1 STI test.

###### Pre-Exposure Prophylaxis Awareness and Willingness

The survey will contain a brief description of PrEP to orient the participant. Most questions were adapted from recent studies of PrEP attitudes with YMSM [38-41]. PrEP awareness will be a single-item measure of whether the participant has heard of PrEP [39]. For participants who do not report current PrEP use, PrEP willingness will be assessed by asking how likely the participant would be to start PrEP in the next 3 months and the reason(s) why the participant is not currently taking PrEP (eg, never heard of PrEP, worried about side effects, lack of support from friends or family).

##### Secondary Outcomes

As secondary outcomes, we will examine the uptake of PrEP, changes in sexual risk behavior, and the linkage and retention in care among newly diagnosed HIV-positive cases. While we expect a small number of newly diagnosed HIV infections, we will measure initiation of antiretroviral therapy and self-reported adherence as a secondary outcome. We are not powered to measure differences in engagement in HIV care across trial arms, so we include this as an exploratory analysis.

###### Mechanisms of Change

Consistent with our theoretical framework, we will assess YMSM’s psychosocial correlates predicting adoption of HIV services (ie, attitudes, norms, self-efficacy, and behavioral intentions to get HIV tested). Integrated Behavioral Model constructs will be assessed with subscales assessing YMSM’s attitudes, social norms, and behavioral intentions [42] that we have used in the past with this population [43]. Social norms assess the extent to which participants feel that friends and family believed the participants should test for HIV. Behavioral intention items assess participants’ intention to adopt HIV testing. Self-efficacy to access HIV or STI services and to discuss sexuality-related issues with partners and provider will be ascertained.

###### Uptake of Pre-Exposure Prophylaxis

At each follow-up assessment, PrEP-eligible (per CDC guidelines), HIV-negative YMSM will be asked whether they have begun using PrEP [39]. YMSM who report using PrEP will be asked to report their adherence to PrEP.

###### Sexual Risk Behavior

Sexual risk behavior will be assessed using the Sexual Practices Assessment Schedule used in previous Web-based studies with YMSM [44,45]. This assessment will explore the number of occasions of different sexual acts (oral, anal; receptive, insertive) with 3 different types of partners (romantic interest, casual partner “hookup,” or friend with benefits), use of condoms during the past 3 months, and knowledge about partners’ HIV status and PrEP use. Assessments ascertain sexual behaviors with male partners and will be conducted at baseline and each follow-up. At-risk sex will be defined as any anal intercourse without condoms or PrEP with a person of known positive and detectable viral load or a person of unknown serostatus during the follow-up period. We will assess the number of partners with whom participants had “at-risk sex,” as well as estimate the incidence of at-risk sex acts (ie, incidence density: the numerator being number of at-risk sex acts and the denominator being person-years of follow time).

###### Linkage and Retention in Care Among Newly Diagnosed HIV-Positive Cases

Among newly diagnosed HIV-positive cases, we will measure participants’ linkage and engagement with appropriate medical care after initial diagnosis, using criteria employed in prior ATN protocols with youth [46-49]. We will define linkage as an HIV-related medical visit within 45 days of referral and engagement as a second HIV-related medical visit within 16 weeks of initial visit [48]. Onset of antiretroviral therapy initiation, self-reported adherence to ART, and viral suppression are exploratory indicators [47], as we recognize that our follow-up period may not be a sufficient amount of time to see these changes.

##### Covariates

We will also measure the following constructs as potential predictors or moderators in our analyses.

###### Sociodemographic Information

We will include questions on participants’ race or ethnicity, educational attainment, employment status, place of birth, housing status, and history of incarceration, sexual identity, and “outness” to their social network.

###### Site Evaluations

Across both trial arms, YMSM who report testing in the prior 3 months will complete site assessments of their testing experiences to measure comfort, quality, and concerns after visiting a site for HIV or STI testing. The site assessment form is the same form used by the mystery shoppers. We will use these assessments to send aggregate data of YMSM’s satisfaction with services to agencies biannually.

###### Substance Use and Psychological Distress

Previous studies have demonstrated higher vulnerability to HIV risk behaviors and engagement in prevention and care among YMSM who report alcohol, tobacco, and other drug (ATOD) use and psychological distress; therefore, we will measure both ATOD and psychological distress as potential effect moderators.

We will assess the frequency of ATOD use (as measured in the National Survey on Drug Use and Health) over the past 3 months in the baseline survey and follow-up surveys for alcohol, tobacco products, marijuana, nonprescription drugs, cocaine, amphetamines, inhalants, opioids (including heroin), hallucinogens, and depressants [50]. If respondents indicate any ATOD use within the past 3 months, we will ask, for each substance, how often the substance was used and if it was used immediately before or during sex.

We will measure psychological distress using existing, well-validated scales: the Patient Health Questionnaire-8 (PHQ-8) [51] and the 7-item Generalized Anxiety Disorder (GAD-7) [52] scale. We will use the first 2 items from each scale to screen participants for symptoms of depression and anxiety (PHQ-2 [53] and GAD-2 [54]). Participants who report depressive symptoms (score of 3 or more on PHQ-2) will be asked the last 6 items from PHQ-8. Participants who report symptoms of anxiety (score of 3 or more on GAD-2) will be asked the last 5 items from GAD-7.

##### Intervention Acceptability

At each follow-up, participants will report on the acceptability of their assigned study arm. We will use the Systems Usability Scale [55] to ascertain participants’ overall satisfaction with the intervention, perception of the information quality, and perceived usefulness of their intervention to improve their health.

###### Use of Intervention Over Time

We will measure intervention exposure using paradata from the intervention, including counts of user sessions, length of sessions, pages visited, and functions utilized. This information will assist in examining whether intervention dosage influences the overall efficacy of the intervention and in informing the cost analysis and wider implementation and scalability [56].

###### Technology and Social Media

We will include Pew Internet survey questions [57] regarding the use of different devices, the number of hours spent online through each device, reasons for social media use, sites commonly frequented, and extent to which the internet supplements face-to-face interactions. We will also measure participants’ frequency of social media use to look for HIV or sexual health-related information [43,58] and their online partner-seeking behaviors [59,60]. We will ask these questions at each follow-up, except for the 1-month follow-up. We will also use the eHealth Literacy Scale [61] to assess participants’ perceived ability to use the internet to find health resources.

#### Statistical Analysis

Descriptive statistics of the psychosocial and demographic characteristics of the participants will be used to describe all participants. These will be compared between treatment groups using *t* tests or Wilcoxon rank sum tests for continuous variables and chi-square tests for categorical variables. To test for intervention efficacy, we will conduct primary analyses of our primary outcomes (HIV testing, STI testing, and PrEP awareness and willingness) using regression analyses to compare our treatment and control groups using the appropriate link function (identity for continuous outcome, logit for binary outcome, and natural log for count outcomes). Interactions between group assignment and these characteristics will be tested to explore the potential moderators of treatment effect. We will repeat these analyses for the secondary outcomes (eg, theoretical mediators, sexual risk behaviors, sexual risk behaviors, PrEP uptake).

We will use the general framework of generalized linear mixed models (GLMM) to test for intervention effects over time. Note that some of our outcomes are binary, some are count, and some continuous traits and thus need to be treated differently. The general form of GLMM will be g(*µ_ij_*) = *β_0i_ + β*_cov_ Covariates*_ij_ + β*_Time_ Times*_j_* + *β*_Arm × Time_, where *µ_ij_* is the mean response corresponding to subject *i* at Time *j (baseline and 4 follow-ups),* with its appropriate link function (identity for continuous outcome, logit for binary outcome, and natural log for count outcomes); *Trt*_i_=1 if the i^th^ subject is in the intervention group, and *Trt*_i_=0 if the i^th^ subject is in the control group. The interaction coefficients *β_Trt×Time_* are of interest here, measuring the difference in the rate of change in outcome across the 2 treatment groups over time. The subject-specific random intercepts *β_0i_* are assumed to be normally distributed with a common variance and they account for within-person correlation. We will also explore if we need a subject-specific random slope corresponding to visit in the above model. Maximum likelihood estimation will be used for fixed effect parameters.

Models will be compared according to the information criteria such as Akaike Information Criterion and Bayesian Information Criterion. For some binary outcomes, such as HIV testing, we will perform an aggregate analysis after collapsing across the repeated measures using simple logistic regression, comparing whether the probability of having tested at least once over the entire follow-up period is different across treatment groups, after adjusting for baseline values. To ensure robustness, we will also apply an exchangeable working correlation structure to its corresponding generalized estimating equation model. We will conduct exploratory regression analyses to examine regional differences. These regressions will be run with group assignment and region in the model, controlling for sociodemographic characteristics. Interactions between group assignment and region will be tested to explore potential site-specific moderators of treatment effect.

As a secondary analysis, we will build on our GLMM framework to examine whether the intervention effects in the theoretical mediators (eg, attitudes, norms, and self-efficacy) are associated with our outcomes. We will also test whether these relationships vary as a function of YMSM’s varying engagement with the intervention (intervention acceptability, use of intervention over time). Interactions between group assignment and these characteristics will test for potential moderators of treatment effect.

#### Cost Analysis

In order to inform the eventual scale-up of GC, we will also conduct a cost analysis of GC and control conditions to inform discussions of sustainability and roll out of the GC intervention. We will collect information on costs associated with the delivery of the intervention. No costs associated with research data collection will be included. These components of cost will be summed over the 12-month study period for each participant to generate an estimated per person cost. Effectiveness will be measured by examining HIV-related outcomes reported by YMSM over the 12-month period. Incremental cost effectiveness ratio (ICER) across treatment arms will be defined as delta C or delta E, where delta C denotes the estimated difference in mean cost of the intervention and delta E reflects the estimated difference in mean effectiveness between the intervention and control groups. Nonparametric bootstrap resampling will be used to estimate the 95% CI of incremental cost effectiveness ratio [62]. Analysis will be performed on participants with complete data. Sensitivity analysis will be conducted by including all participants with multiple imputations for those with missing data.

#### Qualitative Assessment of Testing Sites’ Satisfaction

We will qualitatively assess testing sites’ satisfaction with the biannual performance assessments and their improvements in service delivery when working with YMSM across the 3 regions. Ten site directors will be randomly selected from testing sites in each city. Eligible participants will be able to read and speak English and serve as the site director of an HIV or STI testing site in Philadelphia, Atlanta, or Houston. We will conduct semistructured qualitative in-depth interviews (60-90 minutes) that focus on 4 domains: (1) existing prevention services used and promoted by the agency, (2) agency (internal) resources currently missing, that if identified and addressed, could improve the delivery of HIV, STI, and PrEP services to YMSM, (3) feedback on the biannual performance assessments and their use for service delivery improvements, and (4) the advantages and disadvantages of GC rollout within AIDS Service Organizations.

Interviews will occur via teleconference to maximize candidness and privacy while decreasing travel-related costs. We will use VSee, a simple and low-cost video chat platform that requires no server infrastructure to set up or maintain and allows providers to be HIPAA-compliant. Interviews will be audio-recorded to allow for verbatim transcription, and then checked for accuracy and completion. Initial reading and coding of the transcripts will be reviewed, compared, and refined in team meetings. This systematic process will lead to the creation of a coding structure that includes a hierarchical set of constructs seen in the data. We will analyze several transcripts jointly to establish intercoder reliability. The team will then code all transcripts using our coding structure and add inductive codes during the iterative analysis process. Throughout, we will discuss emerging themes, resolve difficulties or concerns that may arise, and adapt the codebook as necessary.

Since we seek to gain a multilevel understanding of the structural, organizational, and interpersonal barriers and facilitators of implementing GC, our analysis will utilize a phenomenological framework [63]. Although our analysis will rely primarily on a phenomenological inductive approach, we will also employ aspects of deductive analysis that consider our guiding conceptual framework. This combination of analytic strategies will enable us to conduct a phenomenological analysis (inductive) that was initially informed by existing research and theory via the conceptual framework (deductive). We will analyze the qualitative data using thematic analysis until we have reached saturation [64-66].

## Results

GC research activities began in September 2016 and are ongoing. Institutional review board (IRB) submission is complete, with IRB authorization agreements being finalized across the participating universities and SRVs.

## Discussion

There are several potential challenges and limitations to the proposed clinical trial. First, we will rely on self-reported outcomes. We will not include biological measures (eg, presence of HIV or STI), as we would have to dramatically increase our sample size to detect significant effects in biomarkers among newly diagnosed HIV or STI cases and it would be inefficient to collect biomarkers in a Web-based study. We will frame the presentation of results as self-reported outcomes. Second, we propose to recruit a diverse (in terms of race or ethnicity and age) sample of 15-24 year-old participants. It is possible that we may experience more success in recruiting older YMSM (those aged >18 years). To counteract this, we will include a broad range of social media outlets in our recruitment, allowing us the potential to recruit our full age range. Collectively, the team has a vast experience of recruiting youth into HIV research efforts and substantial experience in recruiting online samples of urban race or ethnic minority YMSM. Third, we are unable to untangle race from Latino ethnicity, as it would require a much larger sample size to examine race by ethnicity subgroup differences. Because we propose to quota sample across race or ethnicity in each of the regions, the breakdown of Latino race would create some small sample sizes. Fourth, we recognize that socioeconomically disadvantaged participants may require access to a computer or secure Wi-Fi connection to participate fully in the study. YMSM who are interested in participating but require access or who prefer to complete assessments at a study location will be able to complete intervention activities at their local iTech SRV. Finally, to minimize potential risks, all iTech SRVs have specific policies governing the treatment of human participants, including the referral to medical and psychological services in the event a participant should report a need for these services or experience any adverse reactions resulting from study procedures.

With increasingly promising evidence of the efficacy of biomedical prevention tools, such as PrEP, for reducing the risk of HIV infection among MSM [38,39,67-70], there is increased attention to the potential for HIV testing to act as a gateway to other HIV prevention tools and care efforts [71,72]. Many of the cognitive and behavioral risk factors that contribute to the high rates of HIV infection among MSM are established during adolescence and the transition into young adulthood. This age should be considered a priority time for intervening on cognitive and behavioral risks for HIV, while also introducing YMSM to HIV testing as a gateway to other HIV prevention options.

Efforts to encourage and motivate YMSM to engage in repeat HIV or STI testing or to adopt other prevention efforts (eg, PrEP [38,39,67]) may be diminished if structural barriers (eg, medical mistrust, lack of insurance or transportation) and cultural insensitivity to YMSM’s needs (eg, racial or ethnic and sexual orientation stigma) lead to delays or avoidance of HIV or STI services [10,73,74]. HIV prevention tools must be designed to help YMSM overcome a series of multilevel barriers at the individual (eg, risk awareness), systems (eg, costs, lack of culturally competent care), and structural (eg, homelessness, stigma) levels. Developing strategies to promote the use of HIV prevention services among YMSM requires the creation of interventions such as GC that are culturally sensitive to their psychosocial needs [13] and facilitate access to comprehensive sexual health services [14]. If proven efficacious, GC has the potential to fill a gap in HIV prevention by providing a Web-based, tailored intervention that allows YMSM to learn about local prevention services and to build the skills necessary for successful adoption of prevention.

## Abbreviations

ATOD: alcohol, tobacco, and other drug
CDC: Centers for Disease Control and Prevention
ES: effect sizes
GC: Get Connected
GLMM: generalized linear mixed models
IRB: institutional review board
LGBTQ: lesbian, gay, bisexual, transgender, questioning or queer
MSM: men who have sex with men
PrEP: pre-exposure prophylaxis
RCT: randomized controlled trial
SRV: subject recruitment venues
STI: sexually transmitted infection
YMSM: young men who have sex with men
